# High Serum Squamous Cell Carcinoma Antigen Level Associated with Remission of Mild/Moderate Dysplasia of the Esophagus: A Nested Case–Control Study

**DOI:** 10.1155/2022/2961337

**Published:** 2022-10-13

**Authors:** Hui Su, Kuiliang Liu, Yanjie Zhao, Feng Shi, Yuchen Li, Jiangping Wu, Qingkun Song

**Affiliations:** ^1^Department of Gastroenterology, Beijing Shijitan Hospital, Capital Medical University, Beijing 100038, China; ^2^Department of Gastroenterology, Beijing Friendship Hospital, Capital Medical University, Beijing 100038, China; ^3^Department of Medical Oncology, Beijing Shijitan Hospital, Capital Medical University, Beijing 100038, China; ^4^Department of Pathology, Beijing Shijitan Hospital, Capital Medical University, Beijing 100038, China; ^5^Department of Cell and Molecular Biology, Sid Faithfull Brain Cancer Laboratory, QIMR Berghofer Medical Research Institute, Brisbane, QLD 4006, Australia; ^6^Department of Cancer Research, Beijing Shijitan Hospital, Capital Medical University, Beijing 100038, China; ^7^Department of Clinical Epidemiology, Beijing Youan Hospital, Capital Medical University, Beijing 100069, China; ^8^Department of Clinical Epidemiology, Beijing Shijitan Hospital, Capital Medical University, Beijing 100038, China

## Abstract

**Background:**

The esophageal epithelial dysplasia is the precancerous lesion. This study aimed to investigate the association between the serum squamous cell carcinoma antigen (SCCA) and the remission of esophageal squamous mild or moderate dysplasia.

**Methods:**

We performed a nested case–control study. Patients with mild/moderate dysplasia of the esophageal squamous epithelium were enrolled in this study during the years of 2013–2015 and received a follow-up endoscopy during 2017–2018. With the comparison between baseline and follow-up diagnosis, the patients were divided into regression/stable and progression groups. A predictive model for the outcome of dysplasia was comprised of the variables of SCCA, age, sex, education level, and baseline dysplasia grade. A receiver operating characteristic (ROC) curve was used to estimate the diagnostic efficacy of the regression status of dysplasia under the predictive model.

**Results:**

There were 146 patients enrolled in this study. 100 patients experienced a regression or stable status of dysplasia and 46 patients had a progressed status. Increased age, low education level, and moderate dysplasia were the risk factors of progression. With an 0.1 *μ*g/L increase, SCCA was associated with a 0.90-fold risk (95% CI 0.81, 0.99) of progression. In the predictive model, the area under ROC curve was 0.78. The cut-off values of predictive probability of combined factors for progression, were 0.40 and 0.32 for males and females, respectively.

**Conclusions:**

Increased serum SCCA concentration was associated with regressed severity of mild and moderate dysplasia of the esophageal mucosa. Further studies were warranted and SCCA concentration was a potential biomarker for the dysplasia prognosis.

## 1. Backgrounds

Esophageal cancer ranks 7th in terms of incidence and 6th in terms of mortality globally, with the highest rates in Eastern Asia, where the rates in Mongolia and China are in the top five worldwide [1]. The two most common histologic subtypes are squamous cell carcinoma (SCC) and adenocarcinoma (AC) [2]. In parts of Asia and Africa, SCC accounts for over 90% of all esophageal cancer cases [2]. The major risk factors for SCC are nutritional deficiencies, nitrosamine exposure, and chronic inflammation [3]. The important risk factors for AC include obesity and Barrett's esophagus [1].

Clinical evidence showed that squamous dysplasia is a precancerous lesion of esophageal squamous cell carcinoma (ESCC) in the high-risk population in Linxian, China [4, 5] and the relative risks for developing ESCC were 2.9, 9.8, and 28.3 for individuals with mild, moderate, or severe dysplasia, respectively [5]. Some novel markers for the prognosis of esophageal cancer were explored [6], and besides of esophagectomy, chemotherapy, and radiotherapy, the emerging combined regimen was investigated for the treatment of esophageal cancer [7, 8]. The standard recommendation for managing esophageal squamous dysplasia is based on the histological grades of the disease. Endoscopic resection is recommended to severe dysplasia but the clinical importance of mild and moderate dysplasia is less clear, so clinical observation is recommended to mild and moderate dysplasia. Endoscopic screening with the Lugol dye method, combined with pathologic evaluation, has been recommended to detect early esophageal cancer in the high-incidence areas [9]. However, some people have difficulty tolerating endoscopy because it is an invasive procedure, especially for the asymptomatic cases. Thus, there is an urgent need to build a prognostic model that combines cytological examination, risk factors, and molecular markers for the patients with esophageal squamous mild or moderate dysplasia.

Squamous cell carcinoma antigen (SCCA) is a specific antigen produced by squamous epithelial cells and cancer cells. Upregulation of SCCA could predict early lymph node metastasis [10] and postoperative recurrence [11]. Additionally, the serum SCCA had a positive correlation with the pathologic grade of dysplasia and clinical stage of ESCC [12]. SCCA is an optimal biomarker for the detection of premalignant esophageal lesions [13]. While the prognostic significance of SCCA levels in patients with mild or moderate dysplasia has not been reported. The aim of this nested case–control study is to explore the correlation of serum SCCA concentration, other potential risk factors and the outcome of patients with esophageal squamous cell mild or moderate dysplasia. This study also highlights a prognostic risk model for the vulnerable people who is suffering from this disease.

## 2. Materials and Methods

### 2.1. Ethical Approval

All of the procedures performed in this study involving human participants were approved by the ethical committee of Beijing Shijitan Hospital, Capital Medical University, in accordance with the ethical standards of the 1964 Helsinki declaration and its later amendments. The informed consents were collected from the subjects and the data were anonymized or maintained with confidentiality.

### 2.2. Study Design

A nested case–control study was designed to investigate the correlation between SCCA concentration and the regression of esophageal squamous dysplasia.

### 2.3. Settings

This study was implemented in Yanting County, an area with high incidence rate of ESCC in China. During 2013–2015, local residents aged 40–69, received an endoscopy examination with iodine staining and 10 mL peripheral blood was collected accordingly. The residents with an abnormal iodine staining on the esophageal mucosa under the endoscopy, were prescribed a biopsy. The dysplasia lesions were examined according to an established criteria [14]. During 2017–2018, the selected residents received a follow-up examination of endoscopy with iodine staining on the same mucosa lesions.

### 2.4. Participants

Patients with the diagnosis of mild/moderate dysplasia of the esophageal squamous epithelium were enrolled in our study during the years of 2013–2015. The exclusion criteria included patients with severe liver, kidney, cardiovascular, cerebrovascular, neurological and mental diseases, severe bleeding tendency, prescription of anticoagulant drugs, and iodine allergy.

### 2.5. Variables

Blood samples were collected between 2013 and 2015. Serum SCCA was examined by enzyme linked immunosorbent assay (ELISA; Bioswamp Co., Ltd. Catalog: HM10488). In addition, smoking, drinking, demographic, and dietary indicators were collected at the same time. “Ever smoker” was defined as smoking >100 cigarettes or equivalent use of a pipe over a lifetime [15]. “Ever alcohol drinkers” were defined as individuals who consumed alcohol at least once per month [16]. Family EC history was considered as EC occurrence in the first-degree genetic relatives (parents, siblings, and offspring).

During the period from 2017 to 2018, participants received the follow-up endoscopy with iodine staining. The participants with the mild/moderate dysplasia in the baseline examination regressed to a milder dysplasia lesion or were detected as a consistent dysplasia in a follow-up test were defined as regression or stable disease. The participants progressed to a severer lesion in the follow-up test were defined as progression disease.

The pathological examination and SCCA detection were conducted under the “double blind” mode. Specimens were examined by pathologists to verify tumor types and grade. The inconsistent diagnosis between pathologists were checked by the pathologist with higher qualified position. The baseline metrics were collected before the subjects knew their diagnosis.

### 2.6. Statistical Analysis

All analyses were conducted with SPSS software (version 17.0). The correlation between SCCA concentration and age was analyzed with regression status by the Mann–Whitney *U* test. Dysplasia grade, education level, smoking status, alcohol drinking status, and family cancer history was analyzed with regression status by Chi-square test. Marital status was analyzed with regression status of dysplasia by Fisher exact test. Unconditional logistic regression model was used to analyze the odds ratio (OR) and 95% confidence interval (95% CI) between SCCA level and regression status of dysplasia, with the further adjustments of age, sex, dysplasia grade, and education level. Additionally, the predictive probability of regression status was estimated in the unconditional logistic regression model with the covariates of SCCA, age, sex, dysplasia grade, and education level. A receiver operating characteristic (ROC) curve was used to estimate the area under curve (AUC) between the predictive probability and regression status in males and females, respectively. All analyses were two-sided and the significance level was 0.05.

## 3. Results

One hundred patients had a regression or stable disease and 46 patients had a progressive disease. The patients with regression or stable status had the median age of 66.0, significantly younger than those with progressive disease (Table 1). There was no significant difference between these two groups in terms of sex, marital status, smoking status, alcohol drinking status, or family cancer history (Table 2).

Around 38% patients with regression or stable status had moderate dysplasia, but 54% patients with progressive disease had the moderate dysplasia (*p* = 0.06, Table 1). Nearly 19% patients in regressed or stable status received a junior high school or higher education, compared to 4% patients in progression status (*p* < 0.05, Table 1).

**Figure 1:**
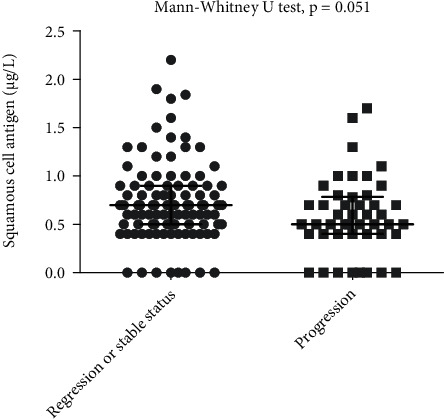
Concentration of SCCA in patients with regression/stable status and progressed status.

The median concentration of SCCA was 0.7 *μ*g/L in patients with regression or stable status, compared to 0.5 *μ*g/L among patients with progressive disease (*p* = 0.05, Figure 1).

In multivariate analysis, OR of 0.1 *μ*g/L SCCA increase was 0.90 (95% CI 0.81, 0.99) (Table 2). Among males, 0.1 *μ*g/L SCCA increase was associated with a 13% reduction in progression risk (95% CI 0.75, 0.99) and the education of junior high school or higher level was associated with 93% risk reduction (95% CI 0.01, 0.67; Table 2). Among females, increase one year of age was associated with a 15% higher risk of progression (OR = 1.15) and the moderate dysplasia had a 359% higher risk of progression (OR = 4.59; Table 2).

**Figure 2:**
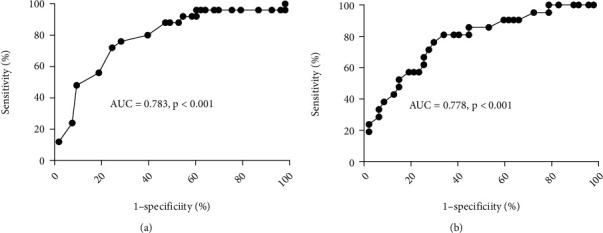
A receiver operating characteristic curve between predictive probability and progression of mild/moderate dysplasia of the esophagus mucosa. (a). Males and (b) females.

In the predictive model for dysplasia progression in males and females, the AUC was 0.78 and 0.78, respectively (*p* < 0.001, Figures 2(a) and 2(b)). In males, the cut-off value of predictive probability was 0.40, with the sensitivity and specificity being 74% and 73%. In females, the cut-off value of predictive probability was 0.32, with the sensitivity and specificity being 76% and 70%.

## 4. Discussion

This study followed up the patients with esophageal squamous cell mild or moderate dysplasia, and revealed that increased concentration of serum SCCA was associated with the reduced risk in progression. Combined with the other risk factors, we generated a model to predict the progression risk for patients with esophageal squamous cell mild or moderate dysplasia. Age and education level were included in this model, patients with older age, lower education level, and moderate dysplasia at baseline had higher risk of progression.

Esophageal squamous dysplasia is the precancerous lesion and the risk of ESCC rises with the severity of dysplasia [5]. In this study, patients with moderate dysplasia had a higher risk of progressing. Approximately 38% patients with regression or stable status had moderate dysplasia, but 54% patients in progression status had the moderate dysplasia at baseline.

The risk factors for esophageal squamous dysplasia have been reported to be similar to those of ESCC [17], including age, family history of cancer, tooth loss, heating stove without chimney, lower socioeconomic status, hot drinking, chronic mucosal irritation, history of aero-digestive tract, and lower educational level [18, 19].

Linzhou city is known as one of the highest incidences of esophageal cancer in China. A case–control study reported that financial status, income, residential space, and education level were all significant risk factors for the cancer [20]. The similar associations had been shown in other studies [21, 22]. In this study, age and education level were included in this predict model. Patients with an older age and a lower educational level have a higher risk of dysplasia progressing.

SCCA2 mRNA expression level in the peripheral blood increased with the severity of esophageal dysplasia, which indicate that SCCA2 mRNA expression in the peripheral blood might be used to monitor premalignant lesions of the esophagus [13]. SCCA was originally isolated from human cervical squamous carcinoma cells and produced by various squamous tumors. The role of SCCA is well investigated in cervical SCC. SCCA was expressed in the suprabasal layers of the stratified squamous epithelium, such as the tongue, tonsil, esophagus, uterine cervix and vagina, Hassall's corpuscles of the thymus, and some areas of the skin. SCCA could be detected in the SCCs of the lung and head and neck. [23] Therefore serum SCCA is widely used as a tumor marker, particularly in detection of SCC [24], and a prognostic marker for cervical cancer [25], and head and neck cancer [26].

SCCA belongs to endogenous serine protease inhibitor family, expressed by malignant cells and normal epithelial cells [27]. It prevents tissue damage from excessive proteolytic enzyme activity due to inflammation [27]. SCCA in normal epithelial acts as an apoptotic inhibitor and promotes the differentiation of squamous epidermis [27]. In this study, we observed an increased level, but within the normal range (0–1.5 *μ*g/L) of SCCA among patients with regressing dysplasia disease. This result possibly indicated that epithelial cells expressed high level of SCCA and suppressed apoptosis effect. But in malignant cells, the expression of SCCA increased to a remarkably high level [27, 28]. The high SCCA in malignant cells inhibited the serine protease and apoptosis, which induced by anti-cancer drugs, TNF-*α* and NK cells [27].

Dysplasia is a premalignant lesion and the increased serum SCCA level may be a protector against apoptosis and prevent the dysplastic lesion progressing to cancer. SCCA was also involved in tumor growth and immune escape. SCCA was at the downstream signaling pathway of interleukin (IL)-4 and IL-13 [29, 30] which prevented the apoptosis induced by IL-2 mediated NK cells [27]. The immune response pathway was involved in the regression and progression of esophageal squamous dysplasia significantly [31]. And the reduction of IL-2 and IL-7 had shown to be associated with regression severity of esophageal dysplasia [32]. Based on the above mechanisms, the increased SCCA among patients experiencing dysplasia regression was probably related to the suppressed apoptosis and altered immune response.

In this study patients with regression or stable status had higher SCCA than that at patients with progression, especially for males. However, the SCCA level in both groups was within the normal range (0–1.5 *μ*g/L). The prognostic model including SCCA, age, sex, education level, and baseline dysplasia grade had an acceptive prediction value. The prediction model comprising of all factors, had consistent diagnosis compacity between male and females. The model was built under the unconditional logistic regression model, which was comparable with the Gail Model for breast cancer risk prediction. But further prospective studies are necessary to validate the prediction efficacy of the model.

Firstly, due to the limitation of laboratory testing conditions, we did not distinguish the subtype of SCCA. Secondly, all the serum samples were collected in 2013–2015 and stored in −70°C refrigerator for more than three years before we tested. Thirdly, SCCA values were generally small and a highly sensitive kit should be used, in case of a considerable amount of clinical error.

In conclusion, increased serum SCCA concentration was associated regressed severity of mild and moderate dysplasia of the esophageal squamous epithelium. SCCA has a great clinical value to be highly recommended used for the progression risk prediction in the vulnerable people.

## Figures and Tables

**Table 1:** The correlation between basic characteristics and regression status.

	Regression or stable disease (*n* = 100)	Progression (*n* = 46)	*p-*Value
Age^a^, median (IQR)	66.0 (8.0)	67.5 (10.0)	0.009
Sex, *n* (%)			0.879
Males	53 (53.0)	25 (54.3)	
Females	47 (7.0)	21 (45.7)	
Dysplasia grade, *n* (%)			0.064
Mild	62 (62.0)	21 (45.7)	
Moderate	38 (38.0)	25 (54.3)	
Marital status^b^, *n* (%)			0.665
Married	95 (95.0)	45 (97.8)	
Others	5 (5.0)	1 (2.2)	
Education level			0.019
Primary school or lower	81 (81.0)	44 (95.7)	
Junior high school or higher	19 (19.0)	2 (4.3)	
Smoking status, *n* (%)			0.349
Never	81 (81.8)	33 (75.0)	
Ever	18 (18.2)	11 (25.0)	
Alcohol drinking status, *n* (%)			0.244
Never	84 (84.8)	33 (76.7)	
Ever	15 (15.2)	10 (23.3)	
Family cancer history, *n* (%)			0.137
No	75 (79.8)	37 (90.2)	
Yes	19 (20.2)	4 (9.8)	

^a^Mann–Whitney *U* test. ^b^Fisher exact test.

**Table 2:** Multivariate analysis in logistic regression model.

	OR^a^	95% CI	*p*-Value	OR^b^	95% CI	*p-*Value	OR^c^	95% CI	*p-*Value
SCC, 0.1 *μ*g/L Increase	0.90	0.81, 0.99	0.039	0.87	0.75, 0.99	0.042	0.97	0.79, 1.19	0.793
Age	1.06	0.99, 1.13	0.063	0.99	0.91, 1.08	0.860	1.15	1.03, 1.30	0.018
Sex			0.178						
Males	1.00	—		—	—	—	—	—	—
Females	0.57	0.25, 1.29		—	—	—	—	—	—
Dysplasia grade			0.146			0.939			
Mild	1.00	—		1.00	—		1.00	—	
Moderate	1.76	0.82, 3.77		1.04	0.35, 3.07		4.59	1.29, 16.39	0.019
Education			0.070			0.021			
Primary school or lower	1.00	—		1.00	—		1.00	—	
Junior high school or higher	0.22	0.04, 1.13		0.07	0.01, 0.67		11.34	0.55, 234.11	0.116

^a^Analysis in all subjects. ^b^Analysis in males. ^c^Analysis in females.

## Data Availability

Data supporting this research article are available from the corresponding author or first author on reasonable request.
